# ASO Author Reflections: Diagnostic Certainty in Characterizing Liver Lesions in Rectal Cancer: Abbreviated Liver MRI Versus CT

**DOI:** 10.1245/s10434-024-16605-x

**Published:** 2025-01-17

**Authors:** Anita Wale, Gina Brown

**Affiliations:** 1 Department of Radiology, St George’s Hospital NHS Foundation Trust, London, UK; 2 Cardiovascular and Genomics Research Institute, St George’s, University of London, London, UK; 3 Department of Surgery and Cancer, Imperial College London, London, UK

## Past

Accurate staging for metastatic disease at diagnosis is crucial to ensure appropriate treatment. Contrast enhanced CT of the thorax, abdomen, and pelvis is routinely performed internationally for patients with colorectal cancer but has suboptimal sensitivity for liver metastases,^1^ and in up to one-third of patients, identifies small liver lesions which are too small to characterise (TSTC).^2^ The resulting diagnostic uncertainty then results in a delay and increased resource use, whilst the patient undergoes gold standard imaging with MRI for formal characterisation of the liver lesions and to exclude metastatic disease.

## Present

We routinely perform abbreviated, non-contrast MRI of the liver at the same time as the initial MRI of the rectum. 42% of patients had a liver lesion identified on CT, compared with 56% by MRI. Six patients had liver metastases at baseline (five diagnosed by CT, the sixth diagnosed only by abbreviated MRI), but 23% of patients had an indeterminate or too small to characterise liver lesion on CT, compared with only 4% on MRI. Diagnostic certainty of the liver findings was therefore achieved in 93% of patients by MRI compared with 45% by ceMDCT (*p* < 0.0001). All liver metastases were in high-risk rectal cancer, OR 17.18 (*p* = 0.06) with a 12.5% conversion rate of TSTC lesions to metastases in high-risk rectal cancer, 0% in low-risk rectal cancer.

## Future

With the increasing complexity of colorectal cancer management strategies, accurate and timely staging is crucial. We have shown that abbreviated MRI of the liver at diagnosis leads to increased diagnostic certainty of too small to characterise liver lesions.^3^ But, in many centres, routine imaging of all patients in this fashion may be unachievable. A risk stratified approach to performing abbreviated liver MRI should be considered given the increased risk of metastatic disease in patients with mrEMVI positive, mrT3c+, or mr tumour deposit positive rectal cancer.^4^ This is the subject of the ongoing phase II study SERENADE.^5^
